# A randomized triple blind trial to assess the effect of an anthelmintic programme for working equids in Morocco

**DOI:** 10.1186/1746-6148-7-1

**Published:** 2011-01-05

**Authors:** Michael A Crane, Khalid Khallaayoune, Claire Scantlebury, Robert M Christley

**Affiliations:** 1 Society for the Protection of Animals Abroad, 14 John St, London. UK. WC1N 2EB; 2 Department of Parasitology, Institut Agronomique et Vétérinaire Hassan II, B.P. 6202, Rabat-Instituts, Morocco; 3 Department of Epidemiology and Population Health, Institute of Infection and Global Health, University of Liverpool, Leahurst Campus, CH64 6QP

## Abstract

**Background:**

Gastro-intestinal parasitism has been identified as a significant cause of disease in working equids in many countries. This randomized triple-blind trial was designed to assess the impact of an anthelmintic treatment programme (using oral ivermectin and fenbendazole) comparing treated and placebo control populations of working donkeys, mules and horses in field conditions in Morocco. In particular, we assessed animal body weight and condition score, together with a questionnaire-based owner evaluation of number of subjective animal health parameters. Faecal worm egg count was also measured.

**Results:**

239 animals completed the full study, 130 in the treatment group and 109 in the control group. Although the average animal weight increased during the study, this change was not significantly different between the two groups. Animals in the treatment group had a significantly lower strongyle worm egg count and increased in body condition score compared to animals in the control group at each examination during the study period. Owners of animals in the treatment group reported improvement in health and work ability and a beneficial effect on pruritus during the early period of the study. These differences in owner perception between treatment groups had disappeared in the latter stages of the study.

**Conclusion:**

This study demonstrated that a routine anthelmintic treatment programme of three treatments annually can have a significant effect on faecal worm egg count. There may be beneficial consequences for the animal health and productivity. Further research on other populations of working equids in different environments would facilitate the objective planning of effective parasite control strategies for specific situations and provide better understanding of the likely clinical benefits of such programmes.

## Background

It has been estimated that there are over 100 million equids working in the developing world compared to 15.5 million in developed countries [1]. These donkeys, mules and horses are used in mainly in agricultural communities for the essential work of field preparation, planting, harvesting and threshing. In addition, they are also used for market transport and the collection of wood and drinking water. In periurban and urban areas equids are used for transport of goods, people and refuse. With over 27 million of these animals in Africa it is not unreasonable to estimate that over 100 million people on the continent are heavily dependent on the working equine for the economic viability of the family unit [2].

It is evident from many studies that these working equids may harbour significant burdens of internal parasites [3-7]. Such parasitic burdens may have significant consequences for the health of these animals [6,8]. In addition it has been suggested that affected animals may have a reduced work capacity [4]. Organizations involved in their care invest significant sums in the administration of anthelmintics. For example, over 50% of the total number of case interventions in the period 2000 to 2005 by the Society for the Protection of Animals and Nature (SPANA) Morocco involved the administration of ivermectin or fenbendazole (data on file).

The current published research is divided over the actual benefits of such antehelmintic treatment programmes. Some studies suggest that there was a positive impact [3,4,9]. Others were less confident and were unable to identify any significant benefit [10,11]. The aim of this randomized triple-blind trial was to assess the efficacy of an anthelmintic treatment programme comparing treated and placebo control populations of working equids in field conditions in Morocco. The principle objectives were to assess the impact of treatment on the animal body weight and condition score and owner perception using a questionnaire focusing on selected animal health criteria. Faecal worm egg counts were also measured although evaluating drug efficacy through changes in faecal egg excretion was not a key objective. The study was conducted over a 12 month period during 2006 and 2007.

## Results

**Figure 1:**
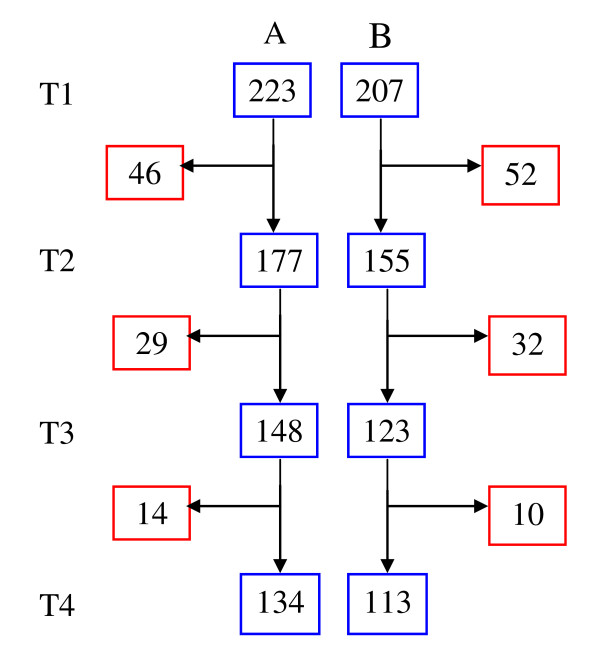
**Number of animals enrolled at each stage of the programme (Blue boxes)**. Red boxes indicate number of animals not returning (8 animals at T4 had incomplete data sets).

Initially, 430 animals were recruited into the study and were presented by their owners at time 1 (T1) (Treatment = 223, Control = 207). Of these, 247 completed the full study period. Of these 239 animals had complete data sets, 130 in the treatment group A (donkey = 35, horse = 49, mule = 46) and 109 in the control group B (donkey = 27, horse = 41, mule = 41). Data on animals initially enrolled in the study but failing to present for all treatments is shown in Figure 1.

In order to evaluate the efficacy of the randomisation process (and the effect of loss to follow-up), variables measured at commencement of the study were compared between the treatment and control groups for animals which completed the study (n = 239). These included age, species, sex, weight, body condition score and faecal worm egg count. No significant differences were observed in any measured variables with the exception of sex. The treatment group contained a greater proportion of males (95/130, 73%) than did the control group (66/109, 61%; p = 0.04). The effect of unequal allocation to treatment group by sex was examined in further analyses and did not affect the results and, for simplicity, was not included in the analyses presented here.

**Figure 2:**
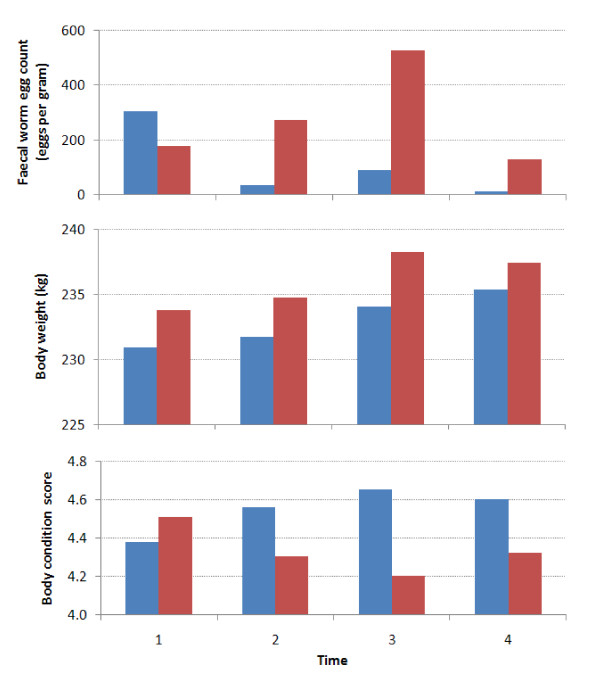
**The impact of treatment with anthelminthics on a) faecal worm egg count, b) body weight and c) body condition score of working mules, donkeys and horses in four regions of Morocco in 2006-2007, based on mixed effects multivariable linear regression models**. Blue bars indicate Treatment A, Red bars indicated Treatment B.

Anthelmintic treatment had a significant impact on faecal strongyle egg count. A significant interaction between treatment group and time was observed (p < 0.001; Table 1 Figure 2a), with the effect of treatment group varying over time. The faecal strongyle egg count was significantly lower in the anthelmintic treatment group compared to the control group at times 2, 3 and 4 (T2, T3 and T4).

**Table 1:** Mixed effects multivariable linear regression models of the impact of treatment with anthelminthics on faecal worm egg count, body weight and body condition score of working mules, donkeys and horses in four regions of Morocco in 2006-2007.

	FWEC^a ^(log_10_+1)			BW^b^			BCS^c^		
	**Coef**	**95% CI^d^**	**p^e^**	**Coef**	**95% CI^d^**	**p^e^**	**Coef**	**95% CI^d^**	**p^e^**

**Random Effects**									
Location	0.35	0.07 to 1.37	0.4	3216.23	592.31 to 12074.14	0.4	0.24	0.02 to 1.18	0.7
Horse	0.39	0.29 to 0.52	< 0.001	4330.21	3612.56 to 518.06	< 0.001	0.57	0.45 to 0.71	< 0.001
Sample	0.89	0.80 to 0.99	< 0.001	209.91	189.17 to 232.96	< 0.001	0.57	0.51 to 0.63	< 0.001
									
**Fixed effects**									0.4
Intercept	2.49	1.97 to 3.07		230.93	178.53 to 284.34		4.38	4.00 to 4.88	
Treatment Group			0.1			0.7			
Treatment	ref								
Control	-0.23	-0.53 to 0.06		2.86	-13.81 to 19.39		0.13	-0.14 to 0.40	
Time			< 0.001			0.05			0.002
1	ref								
2	-0.94	-1.16 to -0.70		0.80	-2.65 to 4.40		0.18	0.00 to 0.36	
3	-0.53	-0.77 to -0.29		3.14	-0.53 to 6.73		0.27	0.09 to 0.45	
4	-1.32	-1.55 to -1.09		4.45	0.91 to 8.03		0.22	0.04 to 0.40	
Treatment × Time interaction			< 0.001			0.9			< 0.001
Control × Time 1	ref								
Control × Time 2	1.12	0.78 to 7.45		0.17	-5.10 to 5.30		-0.38	-0.65 to -0.12	
Control × Time 3	0.99	0.65 to 1.34		1.37	-3.92 to 6.78		-0.58	-0.85 to -0.31	
Control × Time 4	1.18	0.84 to 1.53		-0.78	-6.04 to 4.55		-0.41	-0.67 to -0.14	

^a^FWEC - faecal worm egg count; ^b^BW - body weight; ^c^BCS - body condition score; ^d^CI - confidence interval; ^e^p - p value.

There was no significant difference between the groups in observed weight between the first treatment and subsequent treatments (Table 1 Figure 2b). There was no evidence of an effect of treatment group on animal weight. However, in both the treatment and control groups body weight increased significantly during the trial.

Despite the lack of evidence of an effect on body weight, anthelmintic treatment had a significant impact on body condition score. There was a significant interaction between treatment group and time (p < 0.001; Table 1 Figure 2c) with body condition score being greater in the treated group at times 2, 3 and 4, compared to the control group.

Between T1 and T2 there was a significant difference in the responses of owners of the horses in the two treatment groups with respect to Q1 ("How would you judge the state of health of your animal over the last 2 months?"), Q2 ("How would you assess your animals ability to work during the last 2 months?") and Q6 ("Has your animal suffered from pruritus during the last two months?") (Additional file 1). In each case, owners of animals in the treatment group were more likely than those in the control groups to respond in the more positive categories. This suggests that the treatment group was more likely to result in a perceived improved health, increased ability to work and reduced pruritus in the initial phase of the trial.

Only the reported improved ability to work was still evident when comparing T1 and T 3 (Additional file 1). There was no difference between the groups with regard to perceived health at this stage. Interestingly, owners of horses in the treatment group were more likely to provide a more negative response with regard to pruritus at T3 compared to T1. That is, whilst they often perceived pruritus to have improved between T1 and T2, they were more likely to report a worsening at T3. This effect arises because of those owners in the treatment group that report an improvement in pruritus between T1 and T2 (n = 36), 81% reported a worsening by T3 (11% reported no change and 8% an improvement). In contrast, of those owners in the control group reporting an improvement in pruritus between T1 and T2 (n = 20), 55% reported a worsening by time 3 (45% reported no change and none reported improvement).

If owners responses to all questionnaire items were truly a reflection of their perception of change since the last observation time, these results also suggest that the perceived ability of the horse to work continued to improve between T2 and T3, but that there was no additional improvement in perceived health over this same period.

Comparison of responses for T4 and T1 did not identify significant effects for any of the questions. Hence, by T4 owners in the treatment group were not more or less likely to report an improvement for any of the six health issues, compared to those in the control group.

## Discussion

A limited number of studies have been undertaken to assess the impact on health, performance or body weight of the use of anthelmintics in working equids. This study aimed to investigate these factors.

There was no significant difference between the two groups in body weight between the first and subsequent treatments. A small but significant weight gain was noted in both treatment and control groups over the twelve months of the study. The reasons for this are not known. One possible explanation is that the study started in early summer 2006. The cereal harvest that year was good with straw and grain (the basics of supplementary feeding in Morocco) available at reasonable cost during the later part of the year (data on file). In addition the effect of being enrolled in a trial involving the accurate weighing of animals cannot be discounted. It may have encouraged owners to tend to the needs of their animals and offer them more feed than usual. This possibility that subjects improve that aspect of their behavior being experimentally measured simply in response to the fact that they are being studied has been reviewed [12]. The implication that animals enrolled in the study were adequately fed may influence the interpretation of other results. It has been suggested that in a stressed, undernourished equid internal parasites may have a relatively greater impact on health, the animal being less able to compensate for the parasitic challenge [13].

Despite the lack of evidence of an effect on body weight the treated group had a significantly greater recorded body condition score at T2, T3 and T4 compared to the control group. The authors are unaware of any study that investigates variation in condition score over time in animals with consistent body mass in equids or other species. There are several potential explanations for this apparent effect on body condition. The observed effect may be due to treated animals appearing to be in better health, resulting in a perception of better body condition. In addition, there may be a redistribution of body tissue with treated animals carrying more muscle and fat in those specific body areas assessed in the judgement of condition score. Alternatively, it may indicate that the "blinding" of the personnel assessing condition score was not effective and that they ascribed higher scores to those animals receiving the anthelmintic treatment. However no firm conclusions can be drawn and further investigation of this observation is warranted.

There was a significant reduction in faecal egg counts noted in the treated group at T2, T3 and T4 when compared to the untreated group. This is not unexpected. Small scale studies undertaken in Morocco have demonstrated the efficacy of the products used and no evidence of resistance has been demonstrated (data on file).

Over the course of the year 43% of animals originally enrolled did not complete the full study (Figure 1). There are a number of potential explanations. Experience in Morocco would strongly suggest that the use of animals for timely agricultural tasks or other profitable activity would take precedence over attending to animal health. In addition, animals are traded frequently during their working lives and may be retained only for seasonal activity (data on file).

The importance of helminth parasites and their potentially deleterious impact on working equid health is emphasised by a number of authors [3,4,8,9,14,15]. This potential problem has generally been addressed by the administration of anthelmintics [11]. There are important negative consequences if such treatments are used inappropriately. The potential for development of resistance and environmental concerns dictate that these products should be used correctly and effectively [16]. Additionally, there are significant costs associated with the organisation and administration of treatments to large numbers of animals. It is incumbent on those responsible for such activities that they are undertaken based on objective evidence of their cost/benefit.

The faecal worm egg counts recorded amongst the untreated control animals are lower than those reported in other studies on working equids [4,12,17]. Climatic conditions will influence the lifecycle and risk of grazing working equids acquiring helminth infection [18]. The current study took place during the period June 2006 to July 2007 a period of below average rainfall throughout the study area. This may explain the lower figures for mean faecal worm egg count compared to those of the previous study undertaken in Morocco [4]. It is unlikely that anthelmintic treatment was purchased and administered by the owner or any other animal health practitioner (data on file).

The currently available literature relating to the potential benefits of anthelmintic use in working equids is limited and unclear. Three studies report a measurable benefit from anthelmintic treatment using "body condition scoring" as the sole measure of assessment but have limited information on the methodologies and statistical analyses employed [3,4,9]. In a study that measured body weight, the use of a pre-winter treatment with moxidectin resulted in a 100% reduction in faecal egg counts, improved live weight and body condition score over 16 month period in donkeys kept at a research facility in South Africa [19].

Three studies question the potential benefit of anthelmintic treatment. One study compared animals in Moroccan markets visited by a mobile veterinary clinic which regularly administered anthelmintic treatments with animals in markets not visited [11]. There was no difference in condition score. There was however no record or specific evidence that the animals examined at the "previously treated" markets had in fact received any prior treatment. A further study on pregnant female donkeys and their offspring failed to show any improvement in live weight gain or foal survival when anthelmintic treatment or supplementary feeding was given alone. Only when both were administered did live weight gain in adults and foals and foal survival improve significantly [10]. This study reported changes over a 6 month period. An unpublished study conducted in the north of Ethiopia also questions the benefits of anthelmintic treatment. Compared to controls the results suggest that donkeys treated quarterly with ivermectin failed to show any significant difference in body weight over a twelve month period. (Powell K, personal communication).

The questionnaire was a subjective assessment based on owner perception and memory. Consequently the results and conclusions require future validation. However, decisions regarding therapy, including anthelminthic treatment, are made by owners and as such their perception of the impact of treatment may affect their decision-making.

Cough and colic are common reasons for owners presenting animals at veterinary clinics in Morocco and veterinary practitioners, suspecting parasites as factors in both syndromes, frequently prescribe or administer anthelmintics (data on file). There was no significant difference in the responses to the three questions relating to colic, coughing and diarrhoea and relatively few owners reported signs of "serious colic", frequent coughing, or episodes of diarrhoea.

The perception of a positive impact on "state of health", "ability to work" and "pruritus" is interesting and merits further investigation. Although pruritus was frequently noted no formal attempt has yet been made to diagnose the aetiology of the common diseases in which pruritus is a noticeable sign within this population. Personal observation and discussion with local practitioners would suggest that parasitic dermatoses and Oxyuris equi may be implicated. These results suggest that anthelmintic treatment has a beneficial effect on pruritus, but that this is relatively short lived.

## Conclusions

This study questions the suggestion that for working equids "the one common factor leading to ill health, suffering and early demise is parasitism" [7]. The results presented here suggest that while there may well be benefits that accrue from the use of anthelmintics these benefits may not be clear cut. Furthermore, is perhaps advisable to recall that there may well be different species susceptibility between the horse, the mule and the donkey [13]. Hence any similar intervention needs to be well adapted for the specific situation under consideration.

Further research into the common problems affecting working equids in developing countries is required so that cost effective, appropriate, and sustainable strategies can be developed.

## Methods

The study was conducted in four clinics of the Societe Protectrice des Animaux et de la Nature, Morocco (SPANA) located in Midelt, Khemisett, Had Ouled Frej and Chemaia. These centres have all been established more than ten years and treat predominantly working equine patients (horses, mules and donkeys). The study started (T1) in June 2006 with further treatments in autumn 2006 (T2), spring 2007 (T3) and finished in June/July 2007 (T4).

Initially approximately 100 animals from the local population were enrolled in the study at each centre. Before the first treatment these were checked as being positive on worm egg count and pair matched for species and age. However, on recall for the first treatment it was evident that not all of the enrolled animals would return. It was therefore decided to enroll additional animals and allocate them to the treatment or placebo group, these additional animals being allocated to treatment groups on entry using a pre-prepared random list. Animals were identified by individual brands on the proximal dorsal left fore hoof. In addition, all owners were issued with a laminated identification card.

All participating staff received both collective and individual training. Training sessions covered: weighing technique, hoof branding, questionnaire delivery, coprology, anthelmintic administration, condition scoring, and an overview of the rationale for the study. Training was undertaken by two authors (MC and KK) and all activities were monitored by one author (MC) throughout the duration of the study.

Aging of animals was undertaken by examination of dentition following the guidelines of the American Association of Equine Practitioners [20]. Weighing was undertaken using electronic scales (Ruddweigh Livestock Electronic Weighing Scales, Gallagher, Melbourne, Australia). These were tested using weights of appropriate known mass before and during each session. Condition scoring was undertaken using the 1-9 point scale devised by Pearson and Ouassatt [21].

Faecal worm egg count was undertaken using the Modified Macmaster technique (detection limit 50epg). Faecal samples were stored in a cool insulated box and analysed at the end of each day. Eggs were identified by reference to a standard parasitology text [22]. Only Strongyle-like eggs were counted. However differentiation between Cyathostominae and Strongylinae was not undertaken as this could not be reliably verified.

Six common presenting symptoms or owner complaints for which local veterinary surgeons would frequently prescribe anthelmintic treatment were identified and used to formulate the questionnaire. These were; a history of poor general health, poor work performance, colic, diarrhoea, cough and pruritus. Owners were asked to consider the state of their animal during the preceding two months when responding to the questions. This was done because previous experience in clinical case history taking suggested that whilst owners are aware of the state of health of their animal they are often unable to recall accurately the timing of past events. It was considered that owners would be unable to make a reliable comparison with their animal's pre-study condition. The questionnaire was initially prepared in French, the common language of animal health workers in Morocco. Implementation of the questionnaire was undertaken by the animal technician responsible for each of the four centres. Each technician was able to converse fluently in the appropriate local languages, Moroccan Arabic or Berber, and had a number of years experience in the local area.

The oral anthelminthics used were ivermectin, 0.2 mg/kg (Atlamec, Atlas Veterinaire) and fenbendazole 7.5 mg/ml (Atlafen, Atlas Veterinaire). Ivermectin was used for treatments 1, 2 and 4, fenbendazole for treatment 3. Placebo solutions were formulated to resemble the colour and consistency of the proprietary anthelminthics using solutions of sugar and milk by the author (MC). These were distributed in identical containers to the anthelminthics. The placebo bottles and equipment were labelled "B" and the proprietary anthelminthics "A". Treatment and placebo were referred to simply as "A" and "B" throughout the study. The animal owners and research staff were unaware of the identity of the treatment and control preparations until after completion of the statistical analysis (with the exception of MC, who was not involved in the analysis of data).

Ethical approval for this study was obtained from the Moroccan state veterinary school, The Institute Agronomique et Veterinaire Hassan II. All owners were informed that they would be taking part in a trial to determine the efficacy of an oral medication. Participation in the study was encouraged by the distribution of non-traumatic bits at T2, non-traumatic hobbles at T3 and 15 kg sack of grain at T4. Both the treatment and controls groups received oral ivermectin at T4. No adverse effects were reported in participating animals during the trial period. Two animals from the control group presented with diarrhoea and intermittent colic after receiving oral ivermectin at T4. Both recovered uneventfully with treatment.

## Statistical analysis

Data was managed using Microsoft Access 2007 (Microsoft Corporation, Redmond, WA). In order to assess the efficacy of the randomisation, parameters measured on treatment and control animals at the commencement of the trial were compared using chi-square tests for categorical and Student's t-test for normally distributed continuous variables and the Mann-Whitney U test for non-normally distributed continuous data. Data exploration was conducted in Minitab v15.1 (Minitab Inc., State College, PA).

The effect of treatment group on faecal worm egg count, body weight and body condition score was explored using multilevel multivariable regression models using Markov Chain Monte Carlo (MLwiN v2.02, Rasbach et al, 2000). The number of iterations used was determined by the Raftery-Lewis and Brooks-Draper Nhat statistics [23]. For all models a burn-in of 5000 and chain length of 50,000 was sufficient. Random effects were used to account for clustering within location and within horse. The fixed effects included in the model were; treatment group, time, and a treatment × time interaction. Due to the association between sex and treatment group, sex was also included as a fixed effect. However, this variable did not confound the relationship between treatment group (and time) and the outcomes, and so was not included in the final model. Due to the skewed nature of the faecal worm egg count data, and the presence of zero egg counts, this was transformed (log_10_+1) prior to analysis. Although body condition score was an ordinal categorical variable ranging from 1 to 9, the final model considered this variable to be normally distributed. The fit of each model was assessed by examining the posterior distributions of the fixed and random variables included in the models (data not shown). Following the selected burn-in period and chain length, all fits were smooth and regular and approximated a normal distribution.

In order to investigate the effect of treatment group on owners' responses to questionnaire items, we first looked at the change in response from Time 1 to subsequent sample times. In particular, we considered whether the animal had "worsened", "no change" or "improved" with respect to each questionnaire item. Ordinal logistic regression (Minitab v15.1) was used to investigate whether owners of animals receiving one treatment were more or less likely to report "more positive" responses (i.e. "no change" or "improved") compared to those whose animals received the other treatment. More details of the recoding used for this are displayed in the appendix.

## Abbreviations

epg: eggs per gram; FEC: Faecal Worm Egg Count; T1, T2, T3, T4. These abbreviations refer to the four occasions during the study period when animals were examined and their owners interviewed.

## Authors' contributions

MC participated in the design of the study, monitored the field work and drafted the manuscript. KK advised on the design of the study and trained staff in Morocco. CS and RC carried out the statistical analysis and contributed to writing the manuscript writing. All authors read and approved the final version of the manuscript.

## Supplementary Material

Additional file 1 **Table 2: Ordinal regression models of the impact of treatment with anthelminthics on owner perceived changes in the health of working mules, donkeys and horses in four regions of Morocco in 2006-2007**. Table as described Click here for file
