# Supplementary material for: A randomized triple blind trial to assess the effect of an anthelmintic programme for working equids in Morocco
Source: BMC Vet Res. 2011 Jan 5;7:1. doi: 10.1186/1746-6148-7-1 (PMC3022536; doi:10.1186/1746-6148-7-1)
Supplement: Additional file 1 — Table 2: Ordinal regression models of the impact of treatment with anthelminthics on owner perceived changes in the health of working mules, donkeys and horses in four regions of Morocco in 2006-2007. Table as described [file 1746-6148-7-1-S1.DOC]

**Table 2: Ordinal regression models of the impact of treatment with anthelminthics on owner perceived changes in the health of working mules, donkeys and horses in four regions of Morocco in 2006-2007**

|  | **Time 2 v’s 1** | | | | | **Time 3 v’s 1** | | | | | **Time 4 v’s 1** | | | | |
| --- | --- | --- | --- | --- | --- | --- | --- | --- | --- | --- | --- | --- | --- | --- | --- |
|  | Worsened (row%) | No change (row%) | Improved (row%) | Odds ratio  (95% CI)^1^ | P-value | Worsened (row%) | No change (row%) | Improved (row%) | Odds ratio  (95% CI)^1^ | P-value | Worsened (row%) | No change (row%) | Improved (row%) | Odds ratio  (95% CI)^1^ | P-value |
| **Q1 Health: How would you judge the state of health of your animal over the last 2 months? (possible responses at each time point; worse than normal/as usual/ better than normal)** | | | | | | | | | | | | | | | |
| Treat. | 17 (13) | 68 (53) | 43 (34) | 0.61 (0.37-1.00) | **0.048** | 21 (17) | 61 (48) | 45 (35) | 0.80 (0.49-1.31) | 0.4 | 29 (23) | 62(48) | 37(29) | 1.04 (0.64-1.68) | 0.9 |
| Cont. | 27 (25) | 51 (48) | 29 (27) | ref |  | 22 (21) | 50 (48) | 33 (31) | ref |  | 27(25) | 45(42) | 35(33) | ref |  |
| **Q2 Work: How would you assess your animals ability to work during the last 2 months? (possible responses at each time point; worse than normal/as usual/ better than normal)** | | | | | | | | | | | | | | | |
| Treat. | 9 (7) | 56 (46) | 58 (47) | 0.41 (0.28-0.69) | **0.001** | 7(6) | 62 (50) | 55 (44) | 0.44 (0.27-0.73) | **0.001** | 21(17) | 60(48) | 44(35) | 0.92 (0.56-1.50) | 0.7 |
| Cont. | 22 (21) | 54 (50) | 31 (29) | ref |  | 24 (23) | 49 (46) | 33 (31) | ref |  | 20(19) | 50(47) | 36(34) | ref |  |
| **Q3 Diarrhoea: Has your animal suffered from diarrhoea during the last two months? ( possible responses at each time point; no / yes, occasionally / yes, often)** | | | | | | | | | | | | | | | |
| Treat. | 17 (13) | 94 (74) | 17 (13) | 1.44 (0.81-2.57) | 0.2 | 17 (13) | 93 (73) | 17( 13) | 0.85 (0.48-1.51) | 0.6 | 16(13) | 94(73) | 18(14) | 1.45 (0.82-2.58) | 0.2 |
| Cont. | 10 (9) | 78 (73) | 19 (18) | ref |  | 16 (15) | 78 (74) | 12 (11) | ref |  | 10(9) | 75(71) | 21(20) | ref |  |
| **Q4 Cough: Has your animal had a cough during the last two months? ( possible responses at each time point; no / yes, occasionally / yes, often)** | | | | | | | | | | | | | | | |
| Treat. | 16 (13) | 81 (64) | 30 (23) | 0.94 (0.55-1.60) | 0.8 | 35 (27) | 77 (61) | 15 (12) | 0.99 (0.59-1.67) | 1 | 16(13) | 79(62) | 32(25) | 1.14 (0.67-1.94) | 0.6 |
| Cont. | 11 (10) | 74 (70) | 21 (20) | ref |  | 27 (26) | 68 (65) | 10 (10) | ref |  | 7(7) | 74(70) | 25(24) | ref |  |
| **Q5 Colic: Has your animal suffered from colic during the last two months? ( possible responses at each time point; no / yes, mild or not serious / yes, serious)** | | | | | | | | | | | | | | | |
| Treat. | 6 (5) | 103 (80) | 19 (15) | 0.78 (0.41-1.48) | 0.4 | 22 (17) | 95 (74) | 11 (9) | 1.40 (0.76-2.56) | 0.3 | 7(5) | 99(77) | 22(17) | 0.84 (0.46-1.51) | 0.6 |
| Cont. | 8 (8) | 85 (79) | 14 (13) | Ref |  | 12 (11) | 84 (79) | 10 (9) | Ref |  | 11(10) | 76(72) | 19(18) | Ref |  |
| **Q6 Pruritis : Has your animal suffered from pruritis during the last two months? ( possible responses at each time point; no / yes, mild or not serious / yes, serious)** | | | | | | | | | | | | | | | |
| Treat. | 15 (12) | 77 (60) | 36 (28) | 0.57 (0.34-0.96) | **0.03** | 41 (32) | 69 (54) | 18 (14) | 2.21 (1.32-3.72) | **0.002** | 25(20) | 58(45) | 45(35) | 0.83 (0.51-1.36) | 0.5 |
| Cont. | 21 (20) | 64 (61) | 20 (19) | ref |  | 16 (15) | 66 (62) | 24 (23) | ref |  | 16(15) | 64(60) | 27(25) | ref |  |

**^1^**The odds ratios presented are from Ordinal logistic regression with the lowest category as “worsened”;

OR<1; more likely to be in the upper groups

OR>1; more likely to be in lower groups
